# Biases associated with database structure for COVID-19 detection in X-ray images

**DOI:** 10.1038/s41598-023-30174-1

**Published:** 2023-03-01

**Authors:** Daniel Arias-Garzón, Reinel Tabares-Soto, Joshua Bernal-Salcedo, Gonzalo A. Ruz

**Affiliations:** 1 Departamento de Electrónica y Automatización, Universidad Autónoma de Manizales, Manizales, 170001 Colombia; 2 Facultad de Ingeniería y Ciencias, Universidad Adolfo Ibáñez, 7941169 Santiago, Chile; 3 Center of Applied Ecology and Sustainability (CAPES), 8331150 Santiago, Chile; 4 Data Observatory Foundation, 7941169 Santiago, Chile; 5 Departamento de Sistemas e Informática, Universidad de Caldas, Manizales, 170001 Colombia

**Keywords:** Computer science, Viral infection, Diagnostic markers, Predictive markers

## Abstract

Several artificial intelligence algorithms have been developed for COVID-19-related topics. One that has been common is the COVID-19 diagnosis using chest X-rays, where the eagerness to obtain early results has triggered the construction of a series of datasets where bias management has not been thorough from the point of view of patient information, capture conditions, class imbalance, and careless mixtures of multiple datasets. This paper analyses 19 datasets of COVID-19 chest X-ray images, identifying potential biases. Moreover, computational experiments were conducted using one of the most popular datasets in this domain, which obtains a 96.19% of classification accuracy on the complete dataset. Nevertheless, when evaluated with the ethical tool Aequitas, it fails on all the metrics. Ethical tools enhanced with some distribution and image quality considerations are the keys to developing or choosing a dataset with fewer bias issues. We aim to provide broad research on dataset problems, tools, and suggestions for future dataset developments and COVID-19 applications using chest X-ray images.

## Introduction

Since its first appearance in December 2019 in Wuhan, China^1^, the novel coronavirus became a worldwide pandemic on March 11, 2020^2^. With its exponential growth and the early lack of an effective vaccine or treatment, health professionals focused on the disease’s early diagnosis.

Reverse transcription-polymerase chain reaction (RT-PCR) is the leading diagnosis test for COVID-19. Still, the processing time is long, and the cost is high; enhancing this situation with many tests per day makes the diagnosis slow. Under the circumstances, effective separation of the patients and treatment has no support for early diagnosis^3^. Several studies have shown that chest radiograph (CXR) and computed tomography findings are typical of COVID-19-associated pneumonia^4–6^. For their lower cost compared to computed tomography, X-ray images are valuable assets for COVID-19 recognition (classification) and prognosis (Triage analysis for knowing the best treatment)^7^. Research, in particular, has focused on developing artificial intelligence (AI) models to support the diagnosis of COVID-19 using medical images^8–10^.

The use of AI in diagnosing and triaging suspect COVID-19 patients can enhance the task of distinguishing COVID-19 cases from other cases, even if there is a different type of pneumonia associated. However, some claim it is possible to separate COVID-19 issues from normal ones and ones with bacterial or viral pneumonia. This potential uncertainty could be a limitation for a proper clinical application, considering that the algorithm may not be able to identify the illness, which could lead to false positives or false negative diagnoses. High-accuracy models are shown in the literature, but papers focus on obtaining high-accuracy results but do not consider possible biases present in the datasets used. As a result, some previous articles have studied the bias in datasets related to chest X-ray images in COVID-19^11–13^.

In this paper, we search 46 articles that use AI to detect or triage COVID-19 on X-ray chest images, with accuracy results higher than 90.

As these papers show, COVID-19 datasets were developed in the rise of the pandemic event caused by the COVID-19 spread. Because there were no similar datasets before the sampling process and proper patient selection was rarely even implemented, most of these datasets are images that may contain or not COVID-19 of one or multiple health institutions. Previous work on COVID-19 detection^14^ and the development of datasets using images of S.E.S Hospital de Caldas, showed, after some experiments, that the hospital tests were unsatisfactory, even if training and testing results were high. We saw a bias in terms of the device used for the image capture. An improved dataset that took into account the possible bias and tried to avoid it; has been recently developed^15^.

After researching the metadata provided by some of the most used datasets, we noticed that in terms of patient characteristics and capture conditions are only provided sometimes, or they need to be better structured. Yet, these articles require further studies of the possible biases (and what are the sources of those biases) and show how to measure the bias in a dataset.

With the rise of AI worldwide, many algorithms are proven to contain several types of biases. AI Ethics began to grow as a study topic. Some guidelines have demonstrated effectiveness in regulating AI development and following ethical criteria^16–18^. Following this tendency, we were motivated to develop a two-stage experiment in which we can prove biases, based on medical studies that support that images change (or are affected) depending on the capture conditions and patient characteristics and then make a parity test to test the models using ethical tools and statistical processes. Thus, the contributions of this paper are, first, that we aim not only to provide a more profound argumentation with facts that the datasets have biases but also we conduct a specific experiment on the most used dataset found. Second, we use an ethical tool named Aequitas^19^ that identifies biases in terms of location, sex, and age, among others, on an experiment with a 96.19

The rest of the paper is organized as follows. First, “Databases overview” Section presents the strategy for finding the databases and their corresponding articles. An overview of the database’s information is given in “Bias associated to the datasets” Section.  “Methodology for bias identification in the COVID-19 datasets” Section presents a classification of the types of biases for this problem. Whereas in “Methodology for bias identification in the COVID-19 datasets” section, the methodology for identifying biases present in the COVID-19 databases is described. The experiment using Cohen’s dataset^20^ and the results of the ethical tool are presented in “Discussion” Section. We finish with a discussion of biases and the analysis of the ethical tool in “Conclusion and recommendations” Section, and a conclusion of the study with some recommendations in “Future work” Section.

## Paper search strategy

We search for papers on journal databases such as ScienceDirect, PubMed, IEEE, and Google Scholar and software repositories like GitHub or Kaggle. This search aimed to find documentation related to COVID-19 detection, classification, diagnosis, prognosis, or triage on chest X-ray images (CXR). Detection, classification, and diagnosis can be seen as a classification task where Covid cases are compared with a control group of images. Meanwhile, the triage task consists of comparing positive cases of COVID-19 to know the severity of the affectation and predict future possibilities for the patient. In terms of the excluded articles criteria, we found the following parameters.

- Articles that use another type of data different from Chest X-ray images to classify or triage (Examples CT and other applications of diagnosis validation).
- The dataset used is open. There were only two articles with private datasets and merely for showing that datasets that are not possible have a Bias validation.
- We avoid the review articles.
- In these parts, for most of the papers, we do not consider the article in which the dataset was presented.

We found 46 papers in total; 39 correspond to the classification task^8–10,14,21–55^, 5 to the triage task^56–60^, and two do both tasks^61,62^.

Table 1 Contains the number of subjects in the database, and the metadata of each database, with this they can be associated if there are shared labels within the datasets and if the distributions of the labels are similar to be able to consider the mixture of the datasets to initially avoid biases due to irregular distributions, the references of those papers, and where to download the database. If the database is unavailable, it corresponds to a private database.

**Table 1:** Databases found with the metadata provided, references, and download information.

Database	Subjects	Metadata	References	Download page
Cohen^20^	332	Sex-(Image Amount), Age-(Included), Location-(Europe>Others), Dates-(Included), Others-(ICU admission-Survival model).	^8–10,24–31,55^ ^32–38,41,42,44,45,52–54^	Cohen
BIMCV^63^	4899 positive 5242 negative	Sex-(Patient amount), Age-(Included), Location-(Spain) Dates-(Included),Others-(Classification test).	^14,22,43,46,47,62^	BIMCV-COVID19
Cancer Image Archive^64^	105	Sex-(Image/patient amount), Age-(Included), Location-(EE.UU) Dates-(Not included),Others-(ICU admit-Mortality).	^43,46^	Cancer Image Archive
ML Hannover^65^	71	Sex-(Image amount), Age-(Not included), Location-(Germany) Dates-(Not included),Others-(ICU admission offset-Death offset).	^43^	ML Hannover
BrixIA^57^	2351	Sex-(Image amount), Age-(Included), Location-(Italy) Device-(Included),Dates-(Included).	^23,48,57,62^	BrixIA
Actualmed COVID-19 chest X-rays^66^	216	Sex-(Not included), Age-(Not included) Location-(Spain), Dates-(Not included).	^41,42,48,49^	Actualmed
RYDLS-20^50^	Not mentioned	Sex-(Not included), Age-(Not included) Location-(Not specified), Dates-(Not included).	^50,53^	RYDLS-20
COVID-19 Radiography Database (Qatar university)^67^	Not mentioned	Sex-(Not included), Age-(Not included) Location-(Not specified), Dates-(Not included).	^40–42,45,46,48,49^	Qatar university
SIRM^68^	65	Sex-(Patient amount), Age-(Included) Location-(Italy),Dates-(Not included), Other-(Some symthoms).	^10,60,69^	SIRM
CHUAC dataset^51^	Privated not validated	Sex-(Private not validated),Age-(Private not validated),Location-(Spain).	^51^	Not available for downloading
Radiopaedia^70^	16	Sex-(Patient amount), Age-(Included) Location-(Not mentioned), Dates-(Not mentioned).	^10,69^	Radiopaedia
Eurorad^71^	41	Sex-(Patient amount), Age-(Included) Location-(Europa, America, Asia and Oceania),Dates-(Included).	^10^	Eurorad
AlforCOVID^72^	Not mentioned	Sex-(Image amount), Age-(Included) Location-(Not specified), Dates-(Not mentioned), Other-(ICU admision, Death and Prognosis).	^48^	AlforCOVID
Chest Imaging^73^	50	Sex-(Patient amount), Age-(Included) Location-(Spain), Dates-(Not specified)	^10,60,69^	Chest Imaging COVID-19 CXR Spain
BSTI^74^	40	Sex-(Patient amound), Age-(Included) Location-(UK),Dates-(Included)	^10^	BSTI
Henry Ford Health System^21^	2060	Sex-(Patient amound), Age-(Included) Location-(EE.UU),Dates-(Included)	^21^	Not available for downloading
Figure 1 Covid-19 Chest X ray dataset^75^	48	Sex-(Image amount), Age-(Inlcuded) Location-(Not specified), Dates-(Not included)	^40–42,46,48,49,60^	Figure 1
Covid-QU^76^	Not mentioned	Sex-(Not included), Age-(Not included) Location-(Not specified), Dates-(Not included), Others-(Lungs segmentation mask)	^61^	Covid-QU

## Databases overview

**Figure 1:**
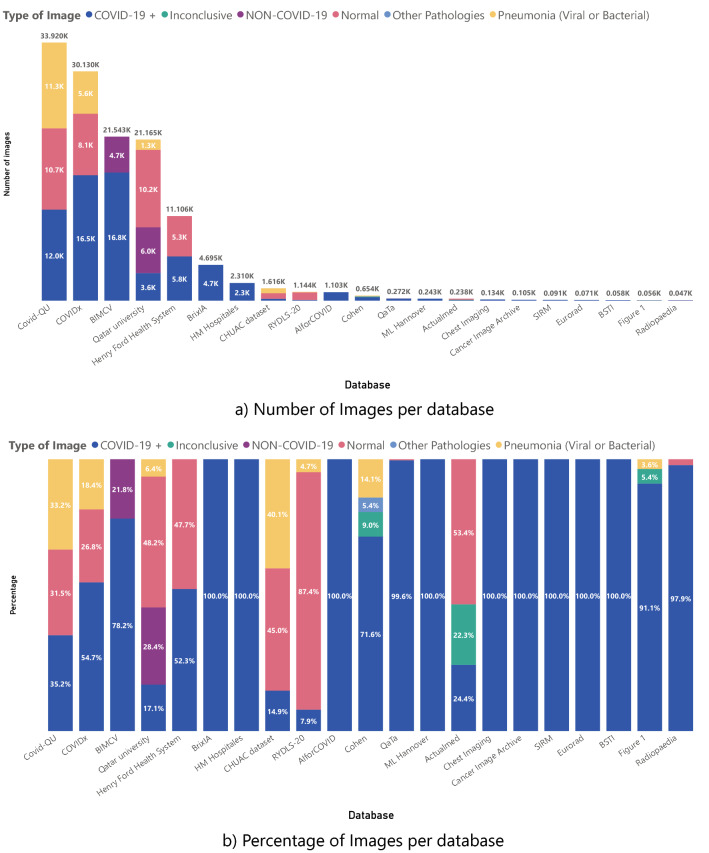
Number and Percentage of images according to a specific pathology on each dataset.

After the search, we obtained 21 datasets, some have parts of the others, and some do not appear in Table 1 because they are a mix of the datasets mentioned. On Mixed datasets is found COVIDx (composed by Cohen, Fig. 1, Actualmed, Qatar University, and an RSNA Pneumonia dataset for control images that appears in Table 2), QaTa (composed by SIRM, Radiopaedia, and Chest Imaging), BrixIA (which contains part of Cohen with a few changes), RSNA, Radiopaedia, and SIRM (small datasets usually used together), COVID-QU (contains QaTa, a Covid GitHub repository, Eurorad, Cohen, SIRM, Qatar University, COVID-CXNet Images, RSNA, Chest X-Ray Images (Pneumonia), and Padchest) which is the largest dataset available found with the addition that every image on the dataset has a lung segmentation mask and RYDLS(Cohen for covid, Radiopaedia for varicella and Mers and Chest X-ray 8 for normal). On the other hand, there are private datasets, which means there is no open access to download them. The datasets in this condition are Henry Ford Health System and CHUAC. Similarly, we could access other available upon request, like BrixIA (to obtain the complete dataset), AlforCOVID, and nd BIMCV. The rest of the datasets are fully open without any requirements.

We believe three main aspects characterize these datasets. First, the size of the datasets, then the type of images, and finally, but not less importantly, the metadata associated with the dataset in general. In terms of dataset size, Fig. 1 shows the number and percentage of types of images in each dataset.

COVID-19 images are validated in two ways, first, by a diagnostic test such as RT-PCR or by validation of characteristics findings by an expert. Other pathologies correspond to any pathology that is not Pneumonia or Covid. Inconclusive cases have no certainty on the diagnosis, or the radiologic report does not contain the associated diagnostic. NON-COVID-19 is a control category that groups images with diverse pathologies and healthy patients. This is mainly used in binary classification so that the models could detect a COVID diagnosis among many possible images, not only of other types of pneumonia or Normal images.

In terms of the content of the datasets, this refers to the image format (normally DICOM or PNG). Image view for area coverage in classification anteroposterior (AP), posteroanterior (PA), or similar views is the leading used, and the lateral test is usually avoided.

**Figure 2:**
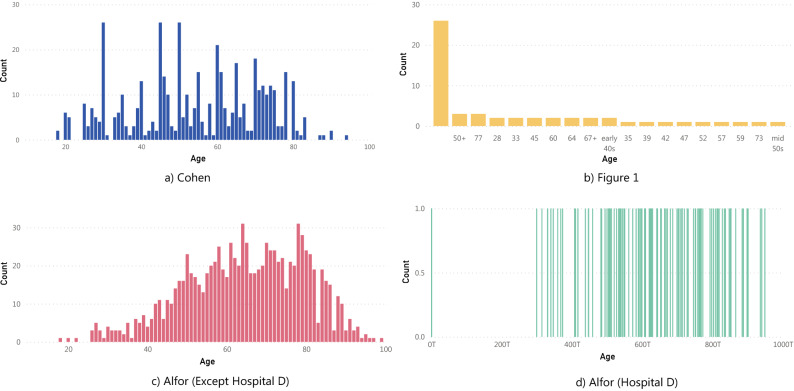
Metadata of some datasets related to the age of the patient with peculiar distributions.

**Figure 3:**
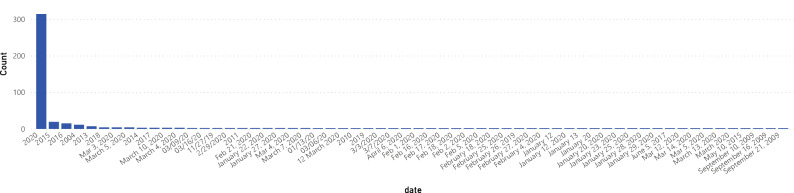
Cohen dataset metadata related to the date of the study (showing inconsistency on this data label).

There are some peculiar distributions on some metadata for the datasets. Figure 2 shows the atypical distribution for Age, and Fig. 3 shows the Cohen Study date distribution that is different from what it should be because the information came from various sources. There are problems with the proper label of the metadata. For more details regarding the metadata of the datasets, Table 1, Figs. 1 and 2 of supplementary material show more complete information on the metadata of each COVID database.

For these datasets, it is important to consider how damaging it can be to mix positive and negative COVID-19 databases. Table 2 shows the databases used for control or to associate with other diseases, with the same information as provided in Table 1.

**Table 2:** Control databases found with the number of papers that use it, references, and where to find the database.

Database	Subjects	Metadata	References	Download page
ChestX-ray 8^77^	30805	Sex-(Images amount), Age-(Included) Location-(No mentioned), Dates-(2017-2020), Others-(Findings)	^10,23,33,44,47,54^	Link
CheXpert^78^	64540	Sex-(Images amount), Age-(Included), Location-(EE.UU) Dates-(2002-2017), Others-(Findings)	^10,35,43,54,62,79^	Link
PadChest^80^	67625	Sex-(Images amount), Age-(Included), Location-(Spain) Dates-(2009-2017), Others-(Findings-symptoms)	^14,22,43^	Link
Chest X-Ray Images(Pneumonia)^81^	5856	Sex-(Not mentioned), Age-(Children) Location-(China), Dates-(Not mentioned)	^8,9,25,27–29,31–34,36–40,45,52,54,55,58^	Link
RSNA PneumoniaDetection Challenge^82^	Not mentioned	Sex-(Not mentioned), Age-(Not mentioned) Location-(China), Dates-(Not mentioned)	^8,30,40–42,46,48,49,52,53,56^	Link
JSRT^83^	Not mentioned	Sex-(Images amount), Age-(Included) Location-(Japan-EE.UU), Dates-(Not mentioned), Others-(Diagnosis)	^28,53^	Link
Montgomey^84^	Not mentioned	Sex-(Images amount), Age-(Included), Location-(EE.UU) Device-(Included), Dates-(Not mentioned), Others-(Findings)	^8,53^	Link
Shenzhen^84^	Not mentioned	Sex-(Images amount), Age-(Included), Location-(China) Deviced-(Included), Dates-(2012), Others-(Findings)	^8,53^	Link

**Figure 4:**
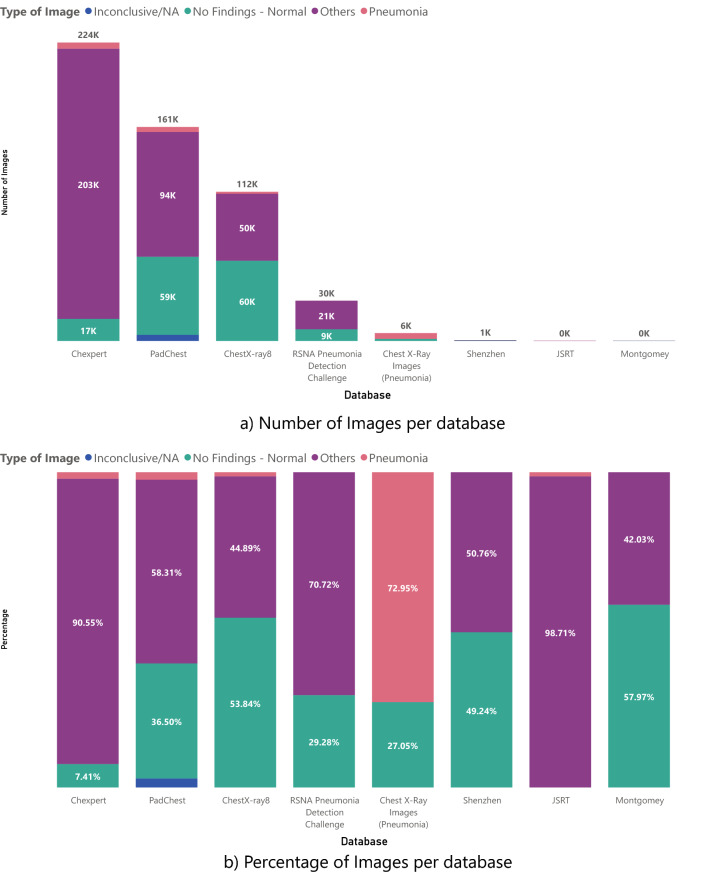
Number and Percentage of images according to a specific pathology on each Control dataset.

As Table 2 Shows that there are eight control image datasets used. Most of these datasets were used for Pneumonia tasks, such as detection and severity or triage tasks. Still, there are also datasets for lung segmentation and simply significant X-ray recollection of different pathologies. There are no private datasets among them, and with a request, we found CheXpert and JSRT. The rest are open, but the RSNA Pneumonia Detection Challenge dataset used in many experiments and as part of the COVIDx dataset is a subset of Chest X-ray 8 and Chest X-ray 8 in its last version is also called Chest X-ray 14. Also, Chest X-ray Images (Pneumonia) is a project mixed with Optical Coherence Tomographies so that it can also be found as “Large Dataset of Labeled Optical Coherence Tomography (OCT) and Chest X-Ray Images”^85^. As on positive ones, Fig. 4 show the percentage and quantity of images associated with specific pathologies.

In terms of Control databases, the metadata associated is sometimes more organized. Still, we also found strange features such as Chest X-Ray images (Pneumonia) and age features that are not specified, but the CXRs are from a pediatric hospital. For more details on Control datasets metadata, follow Table 2 and Fig. 3 of the supplementary material.

## Bias associated to the datasets

Bias in AI can come from many sources, and it is essential to develop equitable systems, generating the lowest bias and relying on ethics tools in AI to prevent models from being racist or sexist, generating problems of discrimination and assumptions that can generate a decrease in the performance of the model, which results in a lack of reliability for the use of these tools. In terms of medical images, many factors can affect the performance of the model generation bias. We want to group by bias associated with COVID-19 Detection or Triage in CXR in four possible groups. Some articles before have shown the possible bias risk in this dataset^86^.

### Bias associated with patient information

People come with different characteristics, and the physical characteristics mainly cause images to look different and that pathologies express differently. Hence, in these cases, we think there are four main reasons that a dataset could have a bias taking into account information of the patients.

- *Sex* The sex can affect CXR mainly because breast tissue in some women is opaque in parts of the images, so datasets with a high number of women compared to men could generate some bias in COVID-19 early stages because the images are initially a little more opaque.
- *Age* The age affects these images in two terms. First, patients with more images are usually old, so the age distribution is generally 60–80 years old. Also, pediatric X-ray chests are not only of the chest (in the image also appears other parts of the patient’s body). Hence, the images differ from the others, and the quantity is considerably less. Age affects the opaque of the images in terms of bone density. Some older people have x-ray findings for some habits like smoking.
- *Patients distribution* The label of patients is super important because in the moment of data splitting for training and testing, using the same patients on both datasets could generate that the model identifies the patients but not the pathology (overfitting).
- *Demographic characteristics* The characteristics of the general population change in every community of the planet, so datasets with many countries in which there are more images from one hospital/city/country than the rest generate that the patient’s characteristics, evolution, and devices used to capture the images change. These changes can cause the model to be biased to recognize images coming from a particular location.

**Figure 5:**
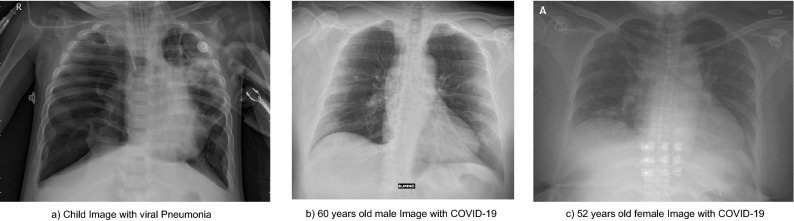
Examples of bias associated with the patient’s information.

For this group of bias, Fig. 5 shows the comparison between a child image from the Chest X-Ray Images (Pneumonia), specifically person_97_virus_180.jpeg, and two adult images (nejmc2001573_f1a.jpeg and 7EF28E12-F628-4BEC-A8C5-E6277C2E4F60.png) of different sex of the Cohen dataset that correspond both to COVID-19 cases.

### Bias associated to capture conditions

The way an image is captured is significant. It can vary much in different devices, hospitals, and countries, so we think three main factors on capturing affect the resulting images, thus, generating bias that may influence the models that use them.

- The device used: many types of devices with different X-ray frequencies affect an image’s shades. Also, portable devices do not have the same quality as normal ones. The unification of this factor is difficult, making the models in many cases biased because the devices used are not the same in COVID-19 images and on the Control dataset, enhanced by the fact that even in the same hospital, if a COVID-19 patient is in the ICU the device must be portable, meanwhile for control cases, the device used is the normal one because there is no limitation on movement.
- Good image capture: even though trained personnel usually take images, some of these could get captured wrong. For example, if the picture is taken on expiration instead of inspiration, the image will be more opaque. If the patient moves in the capture, it could be in a position not suitable for the diagnosis.
- Cables or tubes: if a patient is in the ICU and has ECG cables, nasopharyngeal intubation, and invasive pressure devices, among others, for classifying severity or even COVID-19 from other diseases, the models could learn to identify cables instead of the disease.

**Figure 6:**
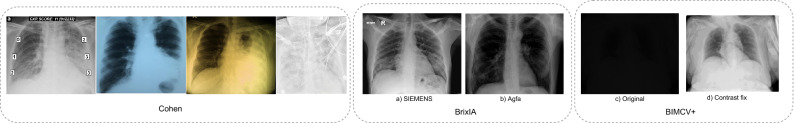
Examples of bias associated to capture conditions in different datasets.

In this case, Fig. 6 shows examples of different capture conditions on the Cohen dataset. It is found that 000005-5-a.jpg is bluer and zoomed than the others, 000012.jpg is yellow, 11547_2020_1202_Fig1_HTML-a.png has labels, and ajr.20.23034.pdf-003.png is whiter than the others and with cables. Also, we found the images 108115246579239728.dcm and 46529543479051320.dcm that are DICOM images from BrixIA that use Siemens and Agfa devices, respectively and for c) and d) sub-S03562_ses-E07248_run-2_bp-chest_vp-ap_cr.png from the BIMCV dataset of a positive case, a 90-year-old male in its original 16 bits PNG format and fixed by changing it with python cv2.IMREAD_GRAYSCALE format.

### Bias associated with unbalanced datasets

Unbalanced datasets are standard in AI problems; in this context, it is not the exception. In particular, COVID-19-positive images are low in comparison with other CXR images. Using it how it is presented may cause the system not to find a remarkable disease pattern. There are two prominent cases to solve this issue. We may balance to the less quantity class, in these cases mainly COVID-19 type that is not suitable because the models will get a small number of images that could affect the model’s generalization power. The other alternative is to use data augmentation for models to have more information. Data augmentation in medical images is quite risky, mainly because some data augmentation processes, such as adding Gaussian noise, may affect the shades on the images and could generate bias on radiological findings associated to a specific pathology different from COVID-19. So the main problem of this approach is the lack of formal clinical validation of the resulting images.

### Bias associated with mixing datasets

Mixing datasets is a standard practice in AI. When only a few COVID-19 images are available (the most common case), mixing with other datasets is helpful because this would be an approach to avoid the unbalanced problem. At the same time, it helps with variability during training allowing the trained model to potentially generalize better. Nevertheless, a common mistake in COVID-19 dataset fusion, mainly on classification tasks, is that by mixing many datasets of COVID-19 and using as Control images another dataset, there will be many variable characteristics on the side of the COVID-19 images. Still, they will be significantly different from the Control images so in the end, most of the time the model will be making differentiation on the dataset used instead of the content of the image. Therefore, in this case, the bias is associated with some past discrimination. There may be people from different places in the COVID-19 dataset in comparison to the Control dataset, and there could be different age or sex distributions.

## Methodology for bias identification in the COVID-19 datasets

In this work, we will analyze biases in the context of COVID-19 classification or diagnosis; other tasks, such as triage, are not considered. Also, we are not assuming that the datasets are deliberately biased, but in terms of the information provided, it is probable to make mistakes that could lead to bias if we were to use it. Finally, if we do not find any data or information about the attributes or a particular attribute, we say it has a bias. For example, suppose the age distribution is not known. In that case, the control dataset could match the COVID-19 dataset. Also, with the patient distribution, even for another task such as triage, if we do not know which images correspond to a specific patient, it is possible to have images of a patient in train and test folds, introducing a bias in the model evaluation.

**Figure 7:**
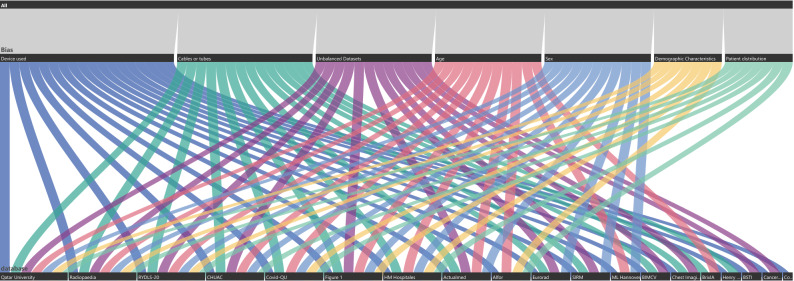
Bias for each COVID-19 dataset.

Figure 7 shows the suggested bias of each COVID-19 dataset, in which we group the bias described before into seven types of bias. Figure 4 of supplementary material shows three of the four groups described in the previous section.

To generate Fig. 7, we took these conditions and clarified each bias label. First, in terms of sex, if one label has more than 70.

Radiopedia is also a particular case among some labels because a study or patient has 31 images apparently from different people. Still, they are all grouped in one Age, Sex, and other information about these images is not provided. Thus, we put this dataset on Age, Sex, and patient distribution bias. Finally, mixed datasets such as RYLDS and Qatar University have unclear demographic information, so they are enclosed in this type of bias. Also, Alfor enters this category because it classifies entities as letters without giving details on the hospital’s locations. Figure 1 is a dataset of images provided by people, so it is difficult to find the place of the images. For the Cables or tubes label, we enclosed the datasets that do not inform if the images belong to a UCI patient. However, if we do not consider these labels in the classification task in the other datasets, it is also a bias in the dataset. Still, we can eliminate those images or perform some cleaning to use those images. Meanwhile, if we do not see each image, it is impossible to identify if a picture contains cables or tubes.

Last but not least, for unbalanced data, if the COVID-19 data is too small or too big, or if the dataset has less than 200 images, it means that the information may not be enough for generalizations, so we classify it as an unbalanced dataset bias.

The following example shows bias associated with mixing datasets. We used the two most popular datasets, Cohen in COVID-19 datasets and Chest X-ray Images (pneumonia), which are also the paramount combination, because eight of the nine papers that use Chest X-ray Images (pneumonia) also use Cohen^8,9,28,34,37,38,45,58^. First, it is essential to see how in Fig. 7 the Cohen dataset only enters in one bias that is the Device used, and also Fig. 3 shows that the Study date has some problems in terms of uniformity; still, it can be handled with some extra work. We notice new biases appear when combining Cohen and Chest X-ray Images (pneumonia) datasets. Deep down on each label sex on Chest X-ray Images (pneumonia) is not found and has more images than Cohen, so the Other label has a higher number of images, which also implies that the dataset is unbalanced because there are 654 images in Cohen. In contrast, 5860 is almost a 9:1 proportion. Also, in terms of sex, we see that the mean Age in Cohen is 54 ± 17 years old, and in Chest X-ray Images (pneumonia), the study uses children, so the mean could be around 11 years old, meaning the age proportion does not match. Also, Cohen contains mainly European images, while Chest X-ray Images (pneumonia) is a Chinese dataset. Finally, even if we subset the Chest X-ray Images (pneumonia) to avoid unbalanced data, age and demographic characteristics bias can not be avoided, and sex can be similar only by luck, so mixing datasets enhances the chances of having bias if the data is not uniform. For a graphical way to show the new bias, the reader can see Fig. 5 of supplementary material.

## Experiment with an ethical tool

As mentioned before, the most used database is the Cohen database. It contains not only COVID-19 cases, so we used this dataset to develop the experiment and its validation with an ethical tool. The ethical tools need metadata associated, so we choose Cohen, which has most of the relevant the less missing data you could validate this in Table 1 of supplementary material. We did not mix it with Chest X-ray (pneumonia) dataset because this last one does not provide any metadata, resulting in the ethical algorithm will fail not because of a bias but instead of a lack of information.

### Experiment

We used only the Cohen dataset and made a few considerations to avoid some bias and focus on the more visible ones according to the metadata’s ethical tool. That includes several labels such as age, sex, location, study date, and if the patient went to ICU, which is vital because of the change in the device used. That leaves the Cohen dataset with two possible biases, the association with mixing the same patient in train and test sets and the unbalanced amount of non-COVID-19 images. To solve this, we executed the following:

- For the division of the images, instead of separating all the images, we split all the patients in the metadata using sklearn.model_selection.train_test_split over the patient amount leaving the training set with 348 patients and 704 images and the test set with 87 patients and 162 images.
- We used sklearn.utils.class_weight to ponder the model weights in the training process for the unbalanced problem.
- Both considerations ensure the model and the dataset can be as much as possible to avoid this type of bias.

Then as a model, we used a pre-trained VGG19 with the Imagenet weights, and as a result, we got a training accuracy of 100

### Ethical tool

For the ethical tool, we used Aequitas^19^. Aequitas is a toolkit to analyze a dataset of AI projects and is available as a web page or desktop program. It also has a Python library^87^ that was also used for this study with a CSV document with a particular structure in which there is a **score** column that corresponds to the binary classification prediction, **label_value** that is the actual class of the classification. A series of attribute columns correspond to categorical strings representing a specific attribute. There are a few recommendations for this format.

- All attribute values have to be a string.
- If the attribute corresponds to a continuous space such as age, we recommend grouping it in intervals.
- The theoretical system works with a high amount of classes on each attribute, but in these case, we found that the optimal amount for a full report and that the graph support all classes is five classes maximum.
- NaN values sometimes are a problem; we suggest group NaN, Blank spaces, and similar in a unique class such as “Not Found” or “Other.”

This tool works with a reference class on each attribute. In our case, we used the class of the higher amount of images, but on the web application, there is an option to select the class automatically by the higher number or, the less bias. The metrics that the tool evaluates are mainly six. These correspond to six types of parity, equality parity, proportionality parity, false-positive rate parity (FPR), false-negative rate parity (FNR), false discovery parity (FDR), and false omission parity (FOR). These metrics can be found in more detail in the Github repository ((https://github.com/BioAITeam/Bias-Covid).

### Results of aequitas

**Figure 8:**
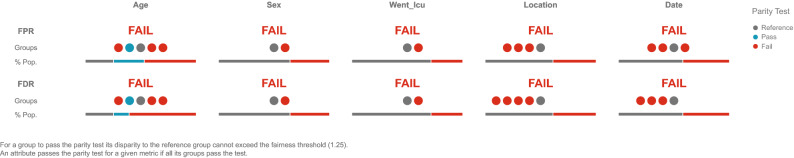
Aequitas results on FPR and FDR for each attribute.

**Figure 9:**
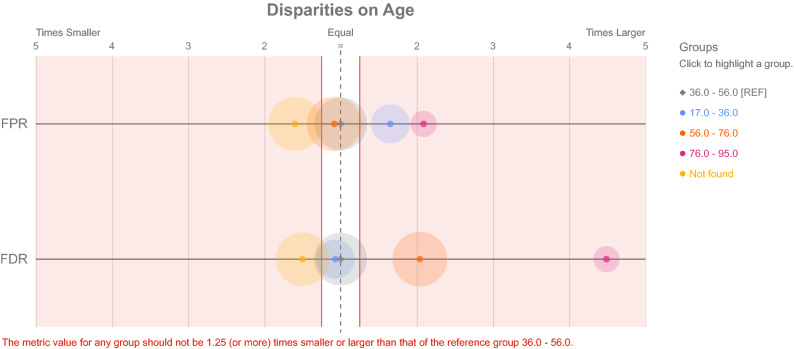
Aequitas results on FPR and FDR the attribute Age.

We used the Python library to find the FPR and FDR on each one of the attributes in Fig. 8; we see results for the test. The red dots are the classes that fail the test, the blue ones are the classes that pass, and the gray one is the reference group; the disparity fairness threshold was set 1.25 times. This graph is dynamic, but in this case, Table 3 contains the information for each class. A deep-down on the Location attribute is in Fig. 9 which it shows how far each attribute is to a parity state.

**Table 3:** FPR and FDR on each attribute detailed to complement Fig. 8.

Attribute	Class	FPR/FDR	Disparity	Parity test
Age	Not found	0.016/0.019	1.60 times smaller in FPR, 1.50 times smaller in FDR	Fail, Fail
Age	56–76	0.023/0.060	1.08 times smaller in FPR, 2.04 times larger in FDP	Pass, Fail
Age	36–56	0.025/0.029	Reference Group	NA
Age	17–36	0.041/0.027	1.65 times larger in FPR, 1.07 times smaller in FDP	Fail, Pass
Age	76–95	0.052/0.133	2.09 times larger in FPR, 4.49 times larger in FDR	Fail, Fail
Sex	M	0.022/0.033	Reference Group	NA
Sex	F	0.039/0.046	1.71 times larger in FPR, 1.41 times larger in FDR	Fail, Fail
Went ICU	Y	0.011/0.019	Reference Group	NA
Went ICU	N	0.051/0.105	4.49 times larger in FPR, 5.53 times larger in FDR	Fail, Fail
Location	Africa	NaN/0.000	5000 times smaller in FDR	Fail
Location	America	0.000/0.000	5000 times smaller in FPR, 5000 times smaller in FDR	Fail, Fail
Location	Asia	0.008/0.033	4.62 times smaller in FPR, 1.70 times smaller in FDR	Fail, Fail
Location	Europe	0.036/0.056	Reference Group	NA
Location	Oceania	0.000/0.000	5000 times smaller in FPR, 5000 times smaller in FDR	Fail, Fail
Date	2000-2019	0.000/0.000	5000 times smaller in FPR, 5000 times smaller in FDR	Fail, Fail
Date	2019-2020	0.000/0.000	5000 times smaller in FPR, 5000 times smaller in FDR	Fail, Fail
Date	2020	0.024/0.095	Reference Group	NA
Date	Not found	0.032/0.020	1.32 times larger in FPR, 4.70 times smaller in FDR	Fail, Fail

In Table 3, all the comparisons are between the respective FPR or FDR of each class with the reference group, which is a division, so if FPR or FDR on a class different from the reference is zero, we will have a zero division so because this relationship trend goes to infinity for the sake of visualization we replace values of zero with 0.0002 because if we use this value tendency, it is 5000 times smaller and 5000 is the maximum value plots, as shown in Fig. 9, could support.

Using the web report generator, we changed some aspects of our attributes. First, we skipped the attribute Date because it contains many metrics that tend to NaN, and we deleted the class Africa from the Location attribute and unite it with the Not Found class. Even though the characteristics are fewer and the parameters on each class. You can find the results of metric failures in the GitHub repository ((https://github.com/BioAITeam/Bias-Covid).

## Discussion

Understanding why these datasets could present each bias is vital to deep-down physiological changes and correlations on patients with particular image characteristics that differ among each dataset. First, if we focus on changes in Chest X-ray images among the age, we find that in the elderly, a reduction in the thickness of the parietal muscles is typical. This generates an increase in pulmonary transparency^88^. This characteristic specifically changes some color range on images. Other characteristics that do not change image transparency but change forms and features of the patients are “barrel chest,” produced by a pronounced dorsal kynopsis and more convexity on the sternum and is common phenotypic in chest elderly, this is typically, but not exclusively, it is also expected on pulmonary emphysema and bronchiectasis, there is also an increase on left ventricular muscles on elderly^88^. But there are not only changes in the elderly if we take into account the development of the respiratory system, we find that the maximum number of alveoli are presented around 10–12 years old, and the maturation of the respiratory system usually ends at 20 years old on females and on 25 years old on males meaning we find less complex pulmonary structures on early stages of life^88^, other common changes are also a decrease on chest wall compliance with the age and dilatation of alveolar duct, so air apace is enlargement with an irregular distribution of air^88^ and as we know the air spaces are fundamental on radio lucid images.

If we focus on pediatric signs and COVID-19 is shown that most symptomatic children with COVID-19 show abnormalities in chest X-rays, but these findings are typically non-specific, so the use of chest x-ray images could not lead to a first diagnostic test for the identification of COVID-19^89^. It is difficult to quality assurance on children’s Chest X-rays. The main factors that affect the quality, especially on young children and babies, are the patient rotation that is inevitable on some babies, the images taken under inspiration because it is challenging to coordinate with the patient respiration timing because you can guide a baby to inspire or expire when you indicate, and finally because of the movement is expected to get a bat scapula position^90^.

Other authors as Albrandt-Salmeron et al.^91^ agree that there is a correlation between age and some symptoms and image findings, but they find out that upon the Mexican-mestizo community, there are no significant different in terms of patient sex^91^, meanwhile Borghesi and Maroldi, 2020 refutes this idea in a study over Italy that finds significantly higher pulmonary insolvents in males than in females^92^, this information can be interpreted in different ways first Mexican study use patients of only one hospital, but they have 1000 chest x-ray images, and Italian study use information of 100 hospitals without specifying the number of images, and both are using the CXR scoring system for COVID-19 pneumonia, proposed by Borghesi and Maroldi of the Italian study^92^ that is also used On BrixIA dataset for the severity label, the information shows two main possibilities as an overview, first using 100 hospitals shows more generalization on the population than only using one, meaning there is a tendency on pulmonary involvement’s grater in males rather than females, but also we can argue that both studies are equally valid, but the differences between the results depends on the population that the study focus on meaning that on Italian or even European population the COVID-19 findings are more usual in males while in Mexico there is not a marked difference.

Some pathologies, especially ground glass densities, are typical in COVID-19 cases but extremely difficult to detect on portable CXR images but easy to catch on CT^93^. Complementing the visualizations in Fig. 6 we saw that the image format affects, and some datasets contained only images in PNG or JPEG format as Cohen that is contaminated with additional elements such as arrows, numbers, or letters different from the ones provided by the device used, that by the way it is also different there are devices that show the letter R for mark the patient right. Still, it can also be a letter D or A, and there are devices that show a P for portable. There are also cases of DICOM format images that depend on the preprocess treatment. Finally, we found formats that try to leave more information available, like the 16 bits PNG images from the BIMCV dataset shown in Fig. 6 and if we see there are visibly different from usual PNG images.

One important thing to highlight is that this study is focused on the bias on COVID-19 classification or diagnosis, so even if most of the databases have a bias on this task, this does not mean they are useless, foremost the main general problem of the datasets presented is that the datasets are created focused on gathering COVID-19 confirmed patients images. Still, a COVID-19 dataset alone can have some powerful uses as triage or prognosis; if there is a certainty that a patient has COVID-19, it is possible that the patient could have a low chance of getting worst, or it is possible that the pneumonia is severe. Identifying these characteristics in less time, a radiologist could identify helps with the patient’s treatment and if the task is to find the probability of a patient going to the ICU or even passing away helps to make strategies to avoid this result. The task mentioned before has less probability to acquire bias because it is not necessary to mix the dataset with others and have mismatches in terms of age distribution and sex, among others, instead even if the dataset is from only one hospital, these could lead to the generation of a helpful software inside this institution. Also, COVID-QU has the highest number of images in the COVID-19 datasets, which could have much bias for classification but is one of the most extensive Lung segmentation datasets available online. The fact that images come from many sources makes that, in these cases, generalization could be better for many formats in chest X-rays for proper lung segmentation and, this way, enhance algorithms in other tasks. BrixIA, for example, has its own severity classification, which groups specific pathologies in certain image zones. The Cohen dataset author recently published a paper that uses this dataset for severity classification^59^.

As a review of the datasets that are best for COVID-19 diagnosis, it is essential to point out that the main datasets used are the ones entirely free that do not even need a request for their usage. Usually, those datasets are the ones with more problems and missing information that could lead to some bias; then let’s avoid the use of control datasets because it is difficult to get a database that matches and generate the least possible amount of conflict in terms of the image characteristics that could lead into a possible bias, the more presented problem is the device used. It is not something we could solve by filtering because there is no information available, and the cable or tubes can be mitigated by removing the ICU patients of both data frames, but this might enhance the unbalance on the dataset, so it is necessary will need clinical validation before the final deployment on the Health institute or organization.

The experiment performed using the Cohen dataset shows different things. First, we see a high accuracy on the dataset in general, meaning the system could differ images from both labels, been zero COVID-19 and one NON-COVID-19, but the Aequitas analysis did not show good results. Let’s suppose that the unbalanced dataset and the patient bias were well avoided; it would be possible to say that Aequitas metrics that depend on balanced datasets could be avoided in attributes such as date because before 2019, there was no COVID-19 so is an inevitable condition if I work with datasets developed before this date. However, still Figs. 8 and 9, and Table 3 shows that in general terms, the dataset fails in all the metrics, even the ones that do not depend on balanced data. There is a particular case that is Age intervals 17–36 and 56–76; on both, there is one metric (FDR and FPR, respectively) that passes the ethical test. Yet, it is one of the two evaluated, meaning it still has issues even though it was the only one to pass. Also, the 5000 times is in real infinitesimal smaller than the reference group meaning these classes are not generating information to generalize, or there are too small that the bias can not be compared, same with Africa on the location that has a NaN value, so is not possible to get a reasonable interpretation of the result. But if we avoid these two cases, those attributes still fail this test. As mentioned before the case that, in general, the metric failed, the detail on Fig. 8 shows that not all classes in all metrics fail. Still, each attribute has more failure metrics, so it generally fails. For more details, see the GitHub repository (https://github.com/BioAITeam/Bias-Covid).

## Conclusion and recommendations

After reviewing the different databases of chest X-rays that are being used to study COVID-19, it is observed that due to the newness of this virus and the eagerness to obtain results in the rapid and early detection of the disease has motivated the release of numerous databases that may have biases such as those associated with: patient information, capture conditions, imbalance, database mixtures, among others. The above can generate high percentages of accuracy in classifying this pathology. Still, when performing an exhaustive analysis of the information, it is found that the AI algorithms may only be calibrated to identify the characteristic features of the disease if the different biases are managed. In addition, the lack of information in the metadata often does not allow a correct choice of the dataset or the identification of different types of biases, especially when mixing highly heterogeneous databases without such information is desired. Therefore, it is recommended to make a deep analysis of the data and its metadata, such as performing a statistical analysis of all the information to be clear about the quality of the database and additionally to perform a visual examination of the information in the case that the type of data used is images, all this to observe possible early biases and try to mitigate them. It is recommended to perform analysis using ethical tools such as Aequitas to ensure that the database does not have biases of age, gender, or race, among others, and thus obtain results with ethical and responsible standards. For the construction and release of new databases in COVID-19 or any other type of problem, it is recommended to consider the experiments and analyses performed in this work to deliver information as homogeneous as possible, where the only difference is, for example, the detection of pathology. Mixing existing databases to increase the volume of information may not be recommended in COVID-19 because it may introduce biases such as those mentioned in this work. Many exposed databases may work well for other problems unrelated to COVID-19. For future work, we propose to perform lung segmentation on existing databases to focus on the disease area of interest to help AI algorithms identify disease-specific features and mitigate potential biases. The identification of COVID-19 using chest X-rays is an area that is still under construction, and there is a long way to go before AI systems can reliably classify the disease. However, building and releasing high-quality databases with as few biases as possible is necessary to achieve that goal.

In this paper, we provide a series of arguments that show some terrible aspects of using and creating Chest X-ray datasets for COVID-19 classification purposes. In addition, we use an AI ethical tool to make a more profound validation of some characteristics in terms of the bias using a simple model and the most used datasets, hoping this could be an example of how further we can validate a model’s performance.

As recommendations for further studies and database creation, it is vital to create homogeneous data. For a hospital, it is almost impossible to acquire the same amount of images from some age groups or sex. Still, at least the use of the device can be homogeneous for positive and control cases, also do not mix positive with control datasets, also validate the results of a group with an expert radiologist, and make detailed metadata of the images for preventing, patient mix in the different sets, and as an optional parameter, please avoid ICU images, the portable devices quality and the severe state of the patients is evident. These algorithms should be guided more in making a fast test to have an initial presumptuous diagnosis and be able to take rapid actions and avoid a rapid patient health deterioration. Making an ICU image is not so helpful because, in this step, the options for treatment are limited. Finally, we recommend using AI ethical tools or frameworks to find possible bias in the model.

## Future work

As a result of this investigation, we are developing a structured dataset taking into account age, sex, and device distribution of positive and negatives cases of COVID-19 for further experiments in terms of classification and the development of software for an actual COVID-19 classification application that has an ethical validation. In this line, some preliminary results can be found here^15^.

## Additional information

The authors affirm no one has a competing financial interest or personal issues that could influence the work developed in this paper. Code and information available on (https://github.com/BioAITeam/Bias-Covid).

## Supplementary Information

Supplementary Figure 1.

Supplementary Figure 2.

Supplementary Figure 3.

Supplementary Figure 4.

Supplementary Figure 5.

Supplementary Table 1.

Supplementary Table 2.

## Data Availability

The datasets analyzed during the current study are available on multiple repositories that can be accessed throw the links on Table 1 and 2 or Data Availability on supplementary material that contains a summary of all articles and links for downloading the public dataset and this paper repository.
