# Supplementary figures and images for: Biases associated with database structure for COVID-19 detection in X-ray images
Source: Sci Rep. 2023 Mar 1;13:3477. doi: 10.1038/s41598-023-30174-1 (PMC9975856; doi:10.1038/s41598-023-30174-1)

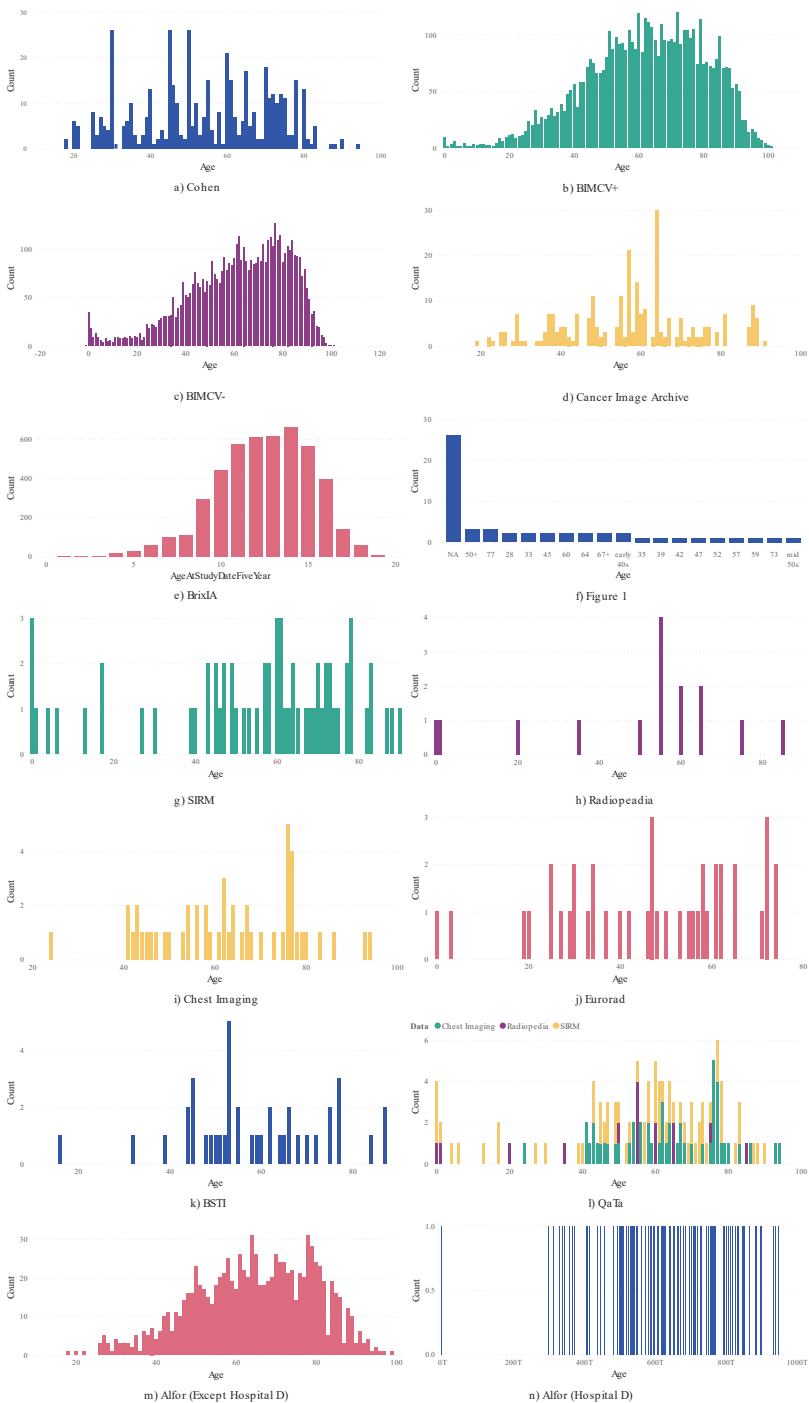

**Figure 1 Supplementary Material: Age distribution from the metadata of the Covid datasets.**

Supplement: Supplementary file 1 — Supplementary Figure 1. [file 41598_2023_30174_MOESM1_ESM.pdf]

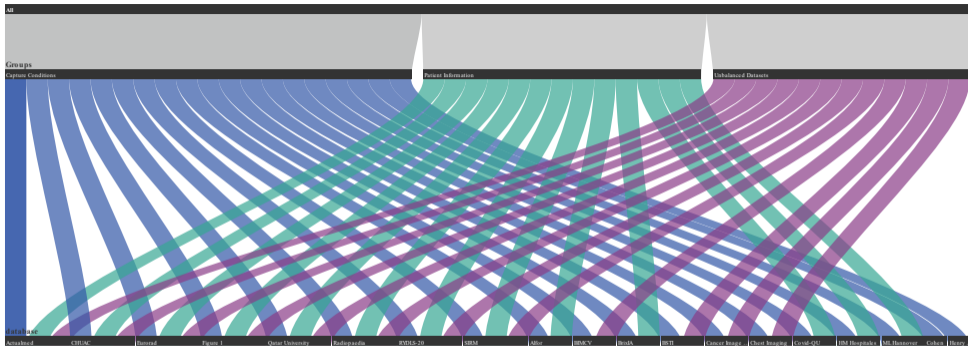

**Figure 4 Supplementary Material:** Groups of bias for each COVID-19 dataset.

Supplement: Supplementary file 4 — Supplementary Figure 4. [file 41598_2023_30174_MOESM4_ESM.pdf]
