# Supplementary material for: Biases associated with database structure for COVID-19 detection in X-ray images
Source: Sci Rep. 2023 Mar 1;13:3477. doi: 10.1038/s41598-023-30174-1 (PMC9975856; doi:10.1038/s41598-023-30174-1)
Supplement: Supplementary file 2 — Supplementary Figure 2. [file 41598_2023_30174_MOESM2_ESM.pdf]

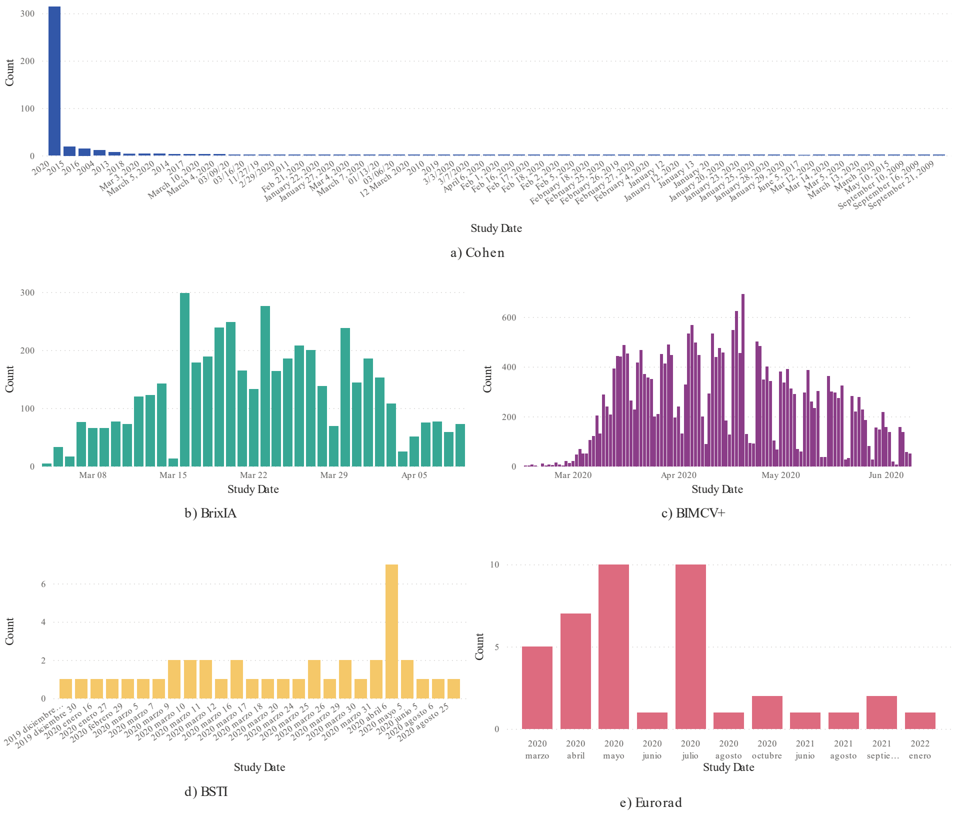

Figure 2 Supplementary Material: Study Dates from the metadata of the Covid datasets.
