# Supplementary material for: Biases associated with database structure for COVID-19 detection in X-ray images
Source: Sci Rep. 2023 Mar 1;13:3477. doi: 10.1038/s41598-023-30174-1 (PMC9975856; doi:10.1038/s41598-023-30174-1)
Supplement: Supplementary file 3 — Supplementary Figure 3. [file 41598_2023_30174_MOESM3_ESM.pdf]

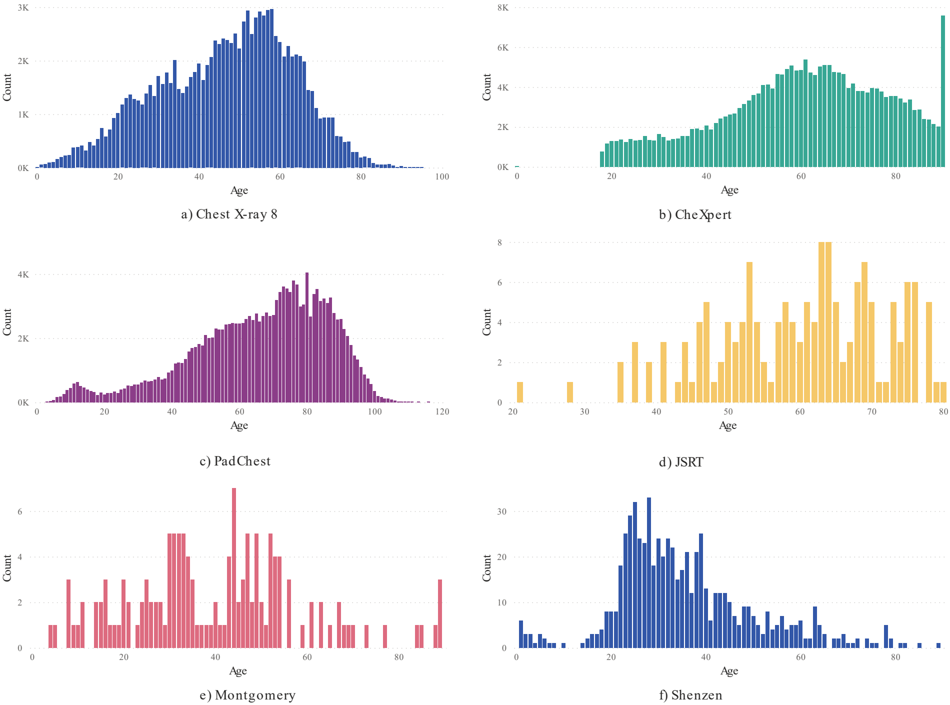


**Figure 3 Supplementary Material:** Age distribution from the metadata of the Control datasets.
