# Supplementary material for: Biases associated with database structure for COVID-19 detection in X-ray images
Source: Sci Rep. 2023 Mar 1;13:3477. doi: 10.1038/s41598-023-30174-1 (PMC9975856; doi:10.1038/s41598-023-30174-1)
Supplement: Supplementary file 5 — Supplementary Figure 5. [file 41598_2023_30174_MOESM5_ESM.pdf]

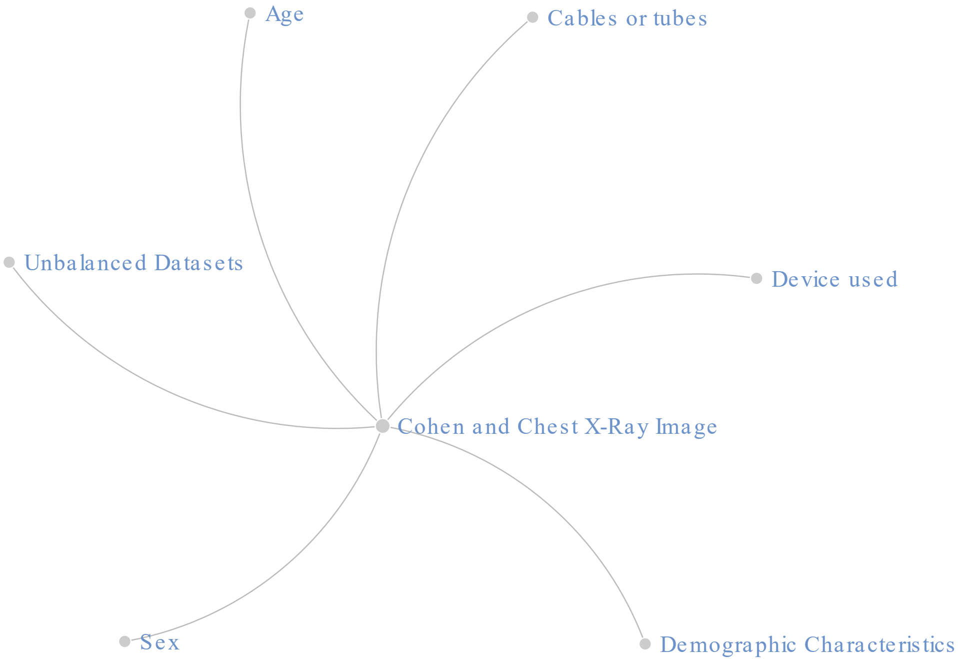

Figure 5 Supplementary Material: Groups of presumed bias for the dataset generated by mixing the Cohen dataset with the Chest X-ray Image (pneumonia) dataset
