# Supplementary material for: Biases associated with database structure for COVID-19 detection in X-ray images
Source: Sci Rep. 2023 Mar 1;13:3477. doi: 10.1038/s41598-023-30174-1 (PMC9975856; doi:10.1038/s41598-023-30174-1)
Supplement: Supplementary file 6 — Supplementary Table 1. [file 41598_2023_30174_MOESM6_ESM.pdf]

Table 1 Supplementary Material: Review of the metadata available on each dataset

Database	Subjects	Sex	Age	Location	Device	Study Dates	Others important
Cohen	332	Images Amount<br>M = 386(60.03%)<br>F = 214(33.28%)<br>O = 43(6.69%)	Fig 3 a)	All around the world but mainly Europe at top with Hannover Medical School, Germany (137 images), italy(71 images), and 43 unkwoun	Not mentioned	Fig4 a)	the ICU Admission And survival label
BIMCV	4899 positive<br>5242 negative	Patient Amount<br>M = 2487(50.77%) + , 2609(49.77%) -<br>F = 2367(48.32%) + , 2592(49.45%) -<br>O = 45(0.92%) + , 41(0.78%) -	Fig 3 b) +<br>Fig 3 c) -	11 Hospitals from the Valencian Region (Spain)	Not mentioned	Fig 4 c)+<br>- not mentioned	Classif cation Test (PCR,IGG, etc) only on Positive dataset
Cancer Image Archive	105	Image/ Patient Amount<br>M = 140(54.69%) / 53(50.48%)<br>F = 116(45.31%) / 52 (49.52%)	Fig 3 d)	United States of America rural area not specif ed	Not mentioned	Not mentioned	ICU Admit and mortality
ML Hannover	71	Image Amount<br>M = 180 (74.07%)<br>F= 63(25.93%)	Not mentioned	Institute for Diagnostic and Interventional Radiology, Hannover Medical School, Hannover, Germany	Not mentioned	Not mentioned	ICU admission of set and Death of set
BrixIA	2351	Image Amount<br>M = 3273(69.71%)<br>F = 1422(30.29%)	Fig 3 e)	the ASST Spedali Civili (Civil Hospital) of Brescia, Italy (ASST-BS) during the pandemic period and some of Cohen dataset	SIEMENS = 2197<br>CARESTREAM HEALTH = 1805<br>AGFA = 553<br>KODAK = 73<br>Villa Sistemi Medicali = 28<br>Agfa-gavaert = 23<br>FUJIFILM = 15<br>DIGITEC = 1	Fig 4 b)	None
HM Hospitales	No information found	No information found	No information found	No information found	No information found	No information found	No information found
Actualmed	216	Not mentioned	Not mentioned	Actualmed, Universitat Jaume I, Spain	Not mentioned	Not mentioned	None
RYDLS-20	Not mentioned	Not mentioned	Not mentioned	Multiple origins as Cohen, radiopedia, but not specif ed	Not mentioned	Not mentioned	None
Qatar university	Not mentioned	Not mentioned	Not mentioned	Multiple origins as SIRM and Chest Imaging not specif ed	Not mention	Not mentioned	None
SIRM	65	Patient Amount<br>M = 41(63.08%)<br>F = 19 (29.23%)<br>O = 5 (7.69%)	Fig 3 g)	Italy dif erent institutions not specif ed	Not mentioned	Not mentioned	Some symthoms
CHUAC dataset	Private not validated	Private not validated	Private not validated	Radiology Service of the Complexo Hospitalario Universitario A Coruña	Private not validated	Private not validated	None
Radiopaedia	16	Patient Amount<br>M = 11 (68.75%)<br>F = 1 (6.25%)<br>O = 4 (25%)	Fig 3 h)	Not mentioned	Not mentioned	Not mentioned	None
Eurorad	41	Patient Amount<br>M = 31(73.61%)<br>F = 10 (24.39%)	Fig 3 j)	11 countries mainly Italy (13), Spain(11), USA(5) and the rest come from Europe, India and Australia	Not mentioned	Fig 4 e)	None
BSTI	40	Patient Amount<br>M = 27 (67.5%)<br>F = 13 (32.5%)	Fig 3 k)	UK patients, not specif ed	Not mentioned	Fig 4 d)	None
Henry Ford Health System	2060	Patient Amount<br>M = 1059 (51.41%)<br>F = 1001 (48.59%)	mean age 62 ± 16 years	[HTML]FFFFFFHenry Ford Health System, which includes f ve hospitals and more than 30 clinics.	Not mention	October to December 2019 not specif ed	None
Figure 1	48	Image Amount<br>M = 11(20%)<br>F = 11(20%)<br>O = 33(60%)	Fig 3 f)	It is an open image collection from people that want to contribute with images, so the origins are diverse and are not specif ed	Not mentioned	Not mentioned	None
Covid-QU	Not mentioned	Not Mentioned	Not mentioned	Multiple origins as BIMCV and Cohen, but there are no metadata in terms of these	Not mentioned	Not mentioned	Lungs Segmentation mask
AIforCOVID	Not mentioned	Image Amount<br>0 = 721 (65.37%)<br>1 = 328 (34.63%)	Fig 3 m) with consideration on Fig 3 n)	6 hospitals marked with letter from A to F with 488 on F hospital the biggest one	Not mentioned	Not mentioned	ICU admission, Death and Prognosis (Mild, Severe)
Chest Imaging	50	Patient Amount<br>M = 31 (62%)<br>F = 19 (38%)	Fig 3 i)	Spain not specif ed institutions or cities	Not mentioned	Not mentioned	None
QaTa	131	Patient Amount<br>M= 83 (63.36%)<br>F = 39 (29.77%)<br>O = 9 (6.87%)	Fig 3 l)	Multiple origins like SIRM and Chest imaging and it is not specif ed	Not mentioned	Not mentioned	None
COVIDx	Not specif ed	Not specif ed	Not specif ed	Multiple origins and with some inside validation make that are not specif ed	Not mentioned	Not mentioned	None
