# Supplementary material for: Biases associated with database structure for COVID-19 detection in X-ray images
Source: Sci Rep. 2023 Mar 1;13:3477. doi: 10.1038/s41598-023-30174-1 (PMC9975856; doi:10.1038/s41598-023-30174-1)
Supplement: Supplementary file 7 — Supplementary Table 2. [file 41598_2023_30174_MOESM7_ESM.pdf]

Table 2 Supplementary Material: Review of the metadata available on each control dataset

Database	Subjects	Sex	Age	Location	Device	Study Dates	Others important
ChestX-ray 8	30805	Images Amount<br>M = 63340(56.49%)<br>F = 48780(43.51%)	Fig 7 a)	Not mentioned	Not mentioned	2017 to 2020<br>not specif ed	Findings
CheXpert	64540	Image Amount<br>M = 132636(59.37%)<br>F = 90777(40.63%)<br>O = 1 (Aprox 0%)	Fig 7 b)	Standford Hospital	Not mentioned	between October 2002 and July 2017, not specifed	14 labels depending on image Findings
PadChest	67625	Image Amount<br>M = 80920(50.3%)<br>F = 79919(49.68%)<br>O = 18(0.01%)	Fig 7 c)	Hospital Universitario de San Juan, Alicante (Spain)	Not mentioned	From January 2009 to December 2017	Many Labels according to Image Findings and symptoms
Chest X-Ray Images (Pneumonia)	5856	Not mentioned	Children not specif c	China various Hospitals not specif c	Not mentioned	Not mentioned	None
RSNA Pneumonia Detection Challenge	Not mention	Not mentioned	Not mentioned	This dataset is a subset from Chest X-ray 8	Not mentioned	2017 to 2020<br>not specif ed	None
JSRT	Not mentioned	Image Amount<br>M = 68 (43.87%)<br>F = 86 (55.48%)<br>O = 1(0.65%)	Fig 7 d)	Healthcare facilities in Japan and the United States not specif ed	Not mentioned	Not mentioned	Diagnosis
Montgomey	Not mentioned	Image Amount<br>M = 63 (45.65%)<br>F = 74 (53.62%)<br>O = 1 (0.72%)	Fig 7 e)	Montgomery County'sTuberculosis screening program, Maryland, USA	Eureka stationary X-ray machine	Not mentioned	Findings
Shenzhen	Not mentioned	Image Amount<br>M = 460 (69.49%)<br>F = 202 (30.51%)	Fig 7 f)	Shenzhen No.3 People's Hospital, Guangdong Medical College, Shenzhen, China.	Philips DR Digital Diagnost system	Within a 1-month period, mostly in September 2012	Findings
